# Changes in Microbial Composition During the Succession of Biological Soil Crusts in Alpine Hulun Buir Sandy Land, China

**DOI:** 10.1007/s00248-024-02359-2

**Published:** 2024-02-16

**Authors:** Yulong Duan, Yuqiang Li, Jianhua Zhao, Junbiao Zhang, Chun Luo, Rongliang Jia, Xinping Liu

**Affiliations:** 1 Key Laboratory of Ecological Safety and Sustainable Development in Arid Lands, Northwest Institute of Eco-Environment and Resources, Chinese Academy of Sciences, Lanzhou, 730000 China; 2 Naiman Desertification Research Station, Northwest Institute of Eco-Environment and Resources, Chinese Academy of Sciences, Tongliao, 028300 China; 3 Shapotou Desert Research and Experiment Station, Northwest Institute of Eco-Environment and Resources, Chinese Academy of Sciences, Zhongwei, 755007 China; 4 University of Chinese Academy of Sciences, Beijing, 100049 China; 5 Key Laboratory of Strategic Mineral Resources of the Upper Yellow River, Ministry of Natural Resources, Lanzhou, 730000 China; 6 Shanghai Majorbio Bio-Pharm Technology Co., Ltd, Shanghai, 200120 China

**Keywords:** Biological soil crusts, Biocrust succession, Desert ecosystems, Hulun Buir Sandy Land, Microbial community

## Abstract

Biological soil crusts (biocrusts) are considered “desert ecosystem engineers” because they play a vital role in the restoration and stability maintenance of deserts, including those cold sandy land ecosystems at high latitudes, which are especially understudied. Microorganisms participate in the formation and succession of biocrusts, contributing to soil properties’ improvement and the stability of soil aggregates, and thus vegetation development. Accordingly, understanding the composition and successional characteristics of microorganisms is a prerequisite for analyzing the ecological functions of biocrusts and related applications. Here, the Hulun Buir Sandy Land region in northeastern China—lying at the highest latitude of any sandy land in the country—was selected for study. Through a field investigation and next-generation sequencing (Illumina MiSeq PE300 Platform), our goal was to assess the shifts in diversity and community composition of soil bacteria and fungi across different stages during the succession of biocrusts in this region, and to uncover the main factors involved in shaping their soil microbial community. The results revealed that the nutrient enrichment capacity of biocrusts for available nitrogen, total nitrogen, total phosphorus, total content of water-soluble salt, available potassium, soil organic matter, and available phosphorus was progressively enhanced by the succession of cyanobacterial crusts to lichen crusts and then to moss crusts. In tandem, soil bacterial diversity increased as biocrust succession proceeded but fungal diversity decreased. A total of 32 bacterial phyla and 11 fungal phyla were identified, these also known to occur in other desert ecosystems. Among those taxa, the relative abundance of *Proteobacteria* and *Cyanobacteria* significantly increased and decreased, respectively, along the cyanobacterial crust–lichen–moss crust successional gradient. However, for *Actinobacteria*, *Chloroflexi*, and *Acidobacteria* their changed relative abundance was significantly hump-shaped, increasing in the shift from cyanobacterial crust to lichen crust, and then decreasing as lichen crust shifted to moss crust. In this process, the improved soil properties effectively enhanced soil bacterial and fungal community composition. Altogether, these findings broaden our understanding about how soil microbial properties can change during the succession of biocrusts in high-latitude, cold sandy land ecosystems.

**Supplementary Information:**

The online version contains supplementary material available at 10.1007/s00248-024-02359-2.

## Introduction

Biological soil crusts (biocrusts) are complex communities formed by microorganisms (bacteria, fungi, archaea), cryptoflora (algae, lichens, moss), and other microscopic organisms bonded to soil surface particles via various secretions, such as mycelium, rhizoides, and polysaccharides [1–3]. As one of the oldest known life forms, biocrusts appeared in the fossil record as early as 2.6 billion years ago [4]. That initial formation of biocrusts long ago is linked to how terrestrial ecosystems originated, in that the widespread development of biocrusts and their improvement of local climate and soil conditions enabled vascular plants to emerge and strongly compete, thereby forming distinct vegetation communities [3]. In this respect, arid and semi-arid ecosystems are particularly noteworthy, since they collectively cover 30–40% of the world’s terrestrial area [5], but in these relatively dry regions, their limited water carrying capacity restricts the viability of large multidimensional vascular plants. Yet, biocrusts are still widely distributed in these ecosystems, constituting at least 70% of their biological cover in some areas, where they effectively enhance soil stability and perform key ecological functions (e.g., providing windbreaks, regulating hydrology, maintaining moisture), as well increasing the fertility and microbial activity of soil [6–9]. Accordingly, biocrusts have earned the moniker “desert ecosystem engineers”, being robust indicators for evaluating the health of desert ecosystems [3, 10, 11].

The morphology and structure of biocrusts is highly diverse, being composed of algae, lichens, and mosses, whose functional types and taxa are mixed together in varying degrees. Although biocrusts count among the planet’s major terrestrial communities, their scientific study started late and early progress was limited. Cyanobacteria, algae, archaea, bacteria, and microfungi are the basic substrates of biocrust formation [12–19], which together promote colonization by lichens, bryophytes, and microorganisms [20–23]. At both global and regional scales, the composition and biomass of particular biocrust communities strongly depends on climatic conditions [24]. For example, for regions whose evapotranspiration potential is relatively high, their biocrusts are mostly composed of low-biomass cyanobacteria, bacteria, and microfungi; i.e., cyanobacterial crusts, lacking mosses or lichens [25, 26]. With declining evapotranspiration, cyanobacteria increase in biomass, lichens and mosses appear, leading to the differentiation and diversification of biocrust types [27, 28]. Beside climate, the soil microhabitat and its characteristics—soil type, texture, nutrient content, salinity, pH, and moisture—can be critical factors shaping the composition and distribution of biocrusts on a regional scale [24, 29].

According to the dominant cryptogam present in them during their succession, biocrusts can be broadly classified into three stages: cyanobacterial crust, lichen crust, and moss crust [30]. As their main biological components, soil microbes (bacteria, fungi, archaea) are collectively responsible for essential ecological functions. Not surprisingly then, trends in the number of dominant species, α-diversity and richness, and community composition of microorganisms across biocrust stages are strongly correlated with biocrust succession [17, 19, 31–33]. Only recently, however, have we begun to explore how the biomass, species composition or ecological roles of these microbial organisms is changed under differing environmental conditions. Technical limitations precluded robust estimates of microbial diversity in previous studies of biocrusts. Fortunately, driven by technological advances in molecular biology within the last decade, microbiome techniques can now be readily applied to reveal the composition of microbial communities at different stages of biocrust succession.

Given the crucial ecological functions of biocrusts in arid and semi-arid ecosystems, in recent years the microbial community composition of biocrusts at different successional stages has been extensively studied in various deserts in distinct bioclimatic zones. This includes *cold deserts* (e.g., Colorado Plateau in the USA) typically found in temperate regions at high elevations, on plateaus, or in mountainous areas, though they also occur in polar regions (Antarctica and Arctic)—that is, generally where the regional mean annual temperature is close to 0 °C [33–37]; *temperate deserts* (e.g., Gurbantunggut Desert and Tengger desert in China, Kyzyl kum desert in Uzbekistan), these usually located at mid-high latitudes, where the regional mean annual temperature is under 18 °C [17, 19, 38–41]; and *hot deserts* (e.g., Atacama Desert in Chile, Namib Desert in Namibia, Negev Desert in Israel), these usually lying at mid-low latitudes and featuring a mean annual temperature above 18 °C, featuring hot summers, daytime temperatures regularly exceeding 30 °C, mild winter climates, and rainfall concentrated in summer (these three desert categories—cold, temperate, and hot—follow the usage at www.britannica.com) [42–47]. Yet, despite recent investigations in several regions, significant knowledge gaps remain concerning the composition of biocrust communities on soils in certain regions, particularly those in underrepresented areas [48, 49].

One of China’s largest sandy land areas is Hulun Buir Sandy Land, lying at the highest latitude among them. According to the above desert classification (www.britannica.com), the Hulun Buir Sandy Land is arguably a *cold desert*. Until now, however, no attempt has been made to investigate the soil microbial community composition of its biocrusts and the drivers of their succession process. Hence, the overarching goal of this study was to apply next-generation-sequencing (NGS) to reveal how the soil microbial community changes during biocrust succession in the Hulun Buir Sandy Land region. Furthermore, considering its high latitude and cold climate, we asked: Could the microbial community composition characteristics and pattern of biocrust succession in this region differ from those in other desert ecosystems? Therefore, our objectives were three-fold: (1) to uncover prominent trends in soil physical and chemical properties during biocrust development and succession; (2) to profile the α- and β-diversity of the soil microbial community vis-à-vis biocrust development and succession; and (3) to elucidate relationships between these complex microbial communities and aspects of their environment. The ecological findings will not only bolster our understanding of biocrusts and their community structure, but are also critical for expanding our knowledge of their diversity and functioning across terrestrial ecosystems.

## Materials and Methods

### Study Site and Soil Sampling

**Fig. 1:**
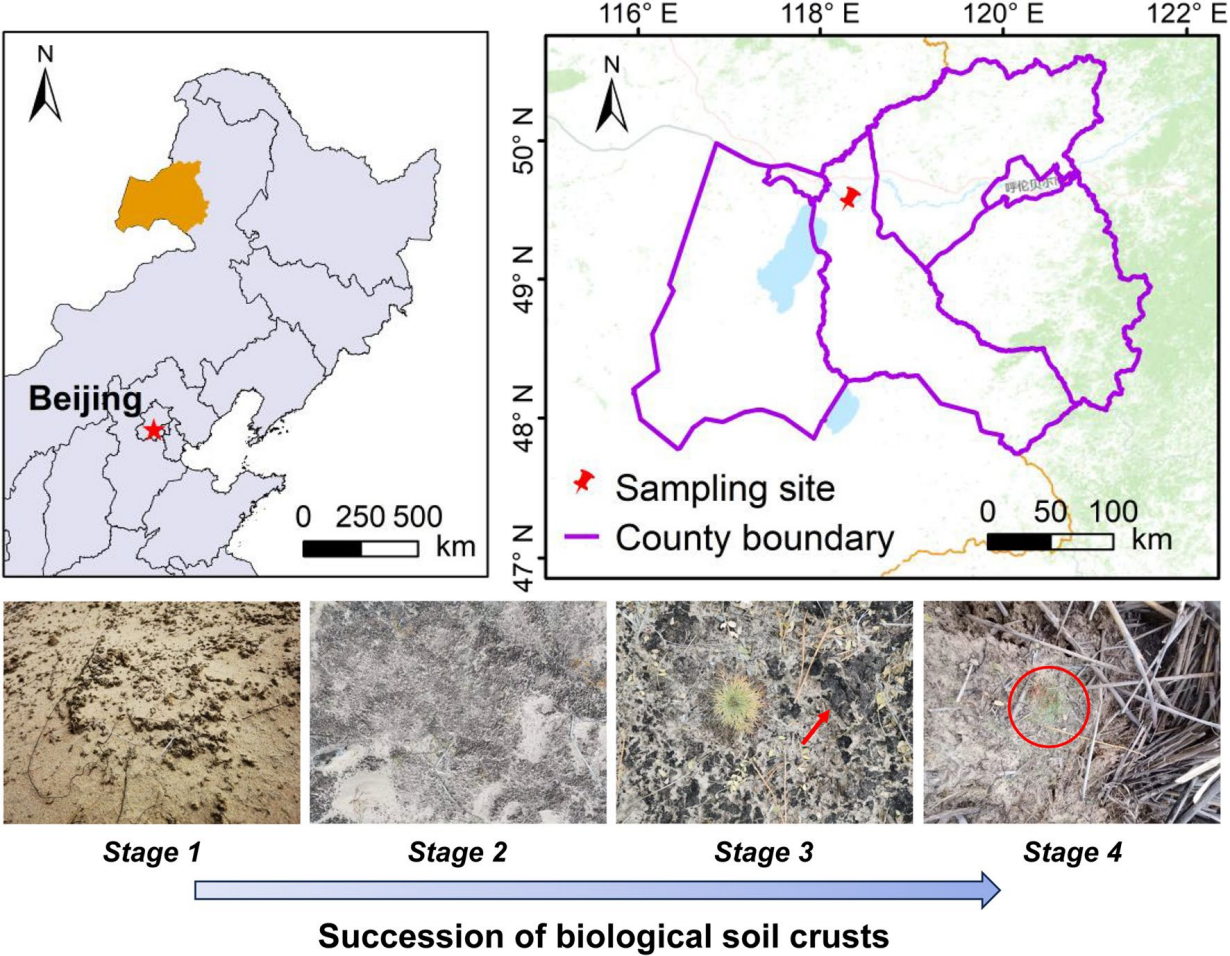
Location of the study site in northeastern China where different stages of biocrust succession in the Hulun Buir Sandy Land were sampled (*n* = 12 interspersed plots). Along the bottom are images of *stage 1*, bare sand (i.e., control, *n* = 3); *stage 2*, cyanobacterial crust (*n* = 3); *stage 3*, lichen crust (*n* = 3); and *stage 4*, moss crust (*n* = 3), which formed the successional gradient investigated (using a ‘space-for-time’ substitution approach). Red arrow and red circle mean lichen crust and moss crust, respectively

The field research was carried out in the New Barag Left Banner of Hulun Buir Sandy Land (Fig. 1), which consists of three large sand belts in northeastern China. Our study site was in the eastern end of the biggest sand belt, which lies along the southern bank of the Hailar River in the northern part of Hulun Buir Sandy Land (118°4′9.7356″ E, 49°19′9.9732″ N, Fig. 1). This region has a temperate continental climate, with a mean annual precipitation (*MAP*) of 290 to 400 mm and a mean annual temperature (*MAT*) of − 5 to 1.5 °C. The zonal vegetation type is typical temperate grassland dominated by annual herbaceous plants, such as *Leymus chinensis*, *Stipa grandis*, *Agropyron cristatum*, and *Carex duriuscula* [50].

In this study, we used a “space-for-time substitution” method to uncover changes in microbial composition during the biocrusts succession in the Hulun Buir Sandy Land in China. At the research site, a total of 12 plots (each 20 m × 20 m) were established, corresponding to four soil cover types: bare sand (no visible crusts, as the control; *stage 1*), cyanobacterial crust (*stage 2*), lichen crust (*stage 3*), and moss crust (*stage 4*) (Fig. 1). Plots having the same biocrust successional stage were separated by more than 1 km. Within each plot, we established four representatives’ subplots (each 5 m × 5 m, at least 10 m apart), in which five soil samples were randomly taken at a 0–5 cm depth under the biological layer, using a sterile cutting ring (9.0-cm diam.). These subplot-level samples were mixed to form one composite sample per plot for each soil cover type, for a total of 12 composite plot-level replicate samples (three × four soil cover types). These were sieved (through 2.0 mm mesh) to remove any visible roots and stones, and then each sample was divided into three portions for further analyses. The first was simply air-dried; the second was stored at 4 °C, to analyze various soil physiochemical properties; immediately after its collection, the third portion was transported on ice to the laboratory where it was freeze-dried at –80 °C for subsequent DNA extractions.

### Soil Physiochemical Properties

For each sample per soil cover type, measurements of its soil material (second portion) were made. Soil particle size composition was determined using a Laser Diffraction Particle Size Analyzer (Mastersizer 2000, Malvern, England). Soil pH of a suspension of soil: water in a 1:5 ratio was recorded with a calibrated pH meter (Mettler Toledo, Giessen, Germany) [51]. To measure the total content of water-soluble salt (*WST*) and soil organic matter (*SOM*), ​we respectively used the residue drying-quality and K_2_Cr_2_O_7_ methods [52]. Total nitrogen (*TN*) was measured using a Kjeldahl analysis system (Kjeltec 8400, Foss, Hillerød, Denmark) [53]. Total phosphorus (*TP*) was determined via colorimetry using sulfuric acid-perchloric acid digestion [54]. Flame photometry was used to quantify total potassium (*TK*), using a perchloric acid-hydrofluoric acid digestion [55]. The soil available nitrogen (*AN*), available phosphorus (*AP*), and available potassium (*AK*) were determined by alkaline hydrolysis diffusion method, molybdenum antimony colorimetry, and flame photometry method, respectively [56].

### Soil DNA Extraction and High-throughput Sequencing

To extract soil DNA from the biocrust samples, the E.Z.N.A. DNA Kit (Omega Bio-Tek, Norcross, GA, USA) was used, by following the manufacturer’s protocols. The obtained nine DNA extracts (three plot-level replicates for each biocrust type (i.e., *stages 2*,* 3*,* 4*) and control (*stage 1*)) were PCR-amplified and then underwent sequencing analyses. The bacterial 16S ribosomal RNA gene and fungal ITS rRNA genes were respectively amplified, using the primer pairs of 338F_806R [57] and ITS1F_ITS2R [58], under these thermocycling parameters: 95 °C for 3 min, followed by 25 cycles at 95 °C for 30 s, 55 °C for 30 s, and 72 °C for 45 s, with a final extension at 72 °C for 10 min. Both PCRs were performed in triplicate, each using a 20-μL reaction mixture containing 2 μL of 5 × FastPfu buffer, 2 μL of 2.5 mM dNTPs, 0.8 μL of each F/R primer (5 μM), 0.2 μL of FastPfu polymerase, and 10 ng of template DNA.

Amplicons were first extracted from 2% agarose gels and purified, using an AxyPrepDNA gel extraction kit (Axygen Biosciences, Union City, CA, USA) according to the manufacturer’s instructions, and then quantified with a QuantiFluor-ST fluorometer (Promega, Madison, WI, USA). Purified amplicons were pooled in equimolar quantities and paired-end sequenced (2 × 300 bp) on the Illumina MiSeq platform PE300. All obtained raw reads have been deposited into the database of the NCBI Sequence Read Archive (SRA) (accession number: PRJNA1026485).

### Bioinformatics Analysis

The paired-end reads from the original DNA fragments were merged using FLASH software [59], a tool designed to combine them when reads 1 and 2 overlap. The resulting paired-end reads were then assigned to each sample according to their unique barcodes. Next, the raw sequencing data were quality-filtered according to these criteria: (i) the reads were truncated at any site that received an average quality score < 20 over a 50-bp sliding window, with those truncated reads < 50 bp removed; and (ii) any reads that had exact barcode matches, or two nucleotide mismatches during primer matching, and which contained ambiguous characters were discarded. Only sequences with overlaps > 10 bp were assembled, this according to their overlap sequence; those reads that could not be assembled were discarded. Operational taxonomic units (OTUs) were clustered with a 97% similarity cutoff using UPARSE [60] and chimeric sequences were identified and removed using UCHIME [61]. Singleton OTUs was removed from the dataset. The taxonomic status of the 16S and ITS rRNA were identified using the RDP Classifier (http://rdp.cme.msu.edu/) against the SILVA (v132) (https://www.arb-silva.de/) or Unite (v8.0) (https://unite.ut.ee/) [62] database, respectively, at a 0.7-confidence threshold. Importantly, to account for differences in their sequencing depth, all samples were normalized in QIIME software (v1.8.0) [63]. The ensuing OTUs were used to calculate the α-diversity and β-diversity metrics.

### Statistical Analysis

The α-diversity (http://www.mothur.org/wiki/Calculators) indexes were calculated using the diversity function of the “vegan” package (https://CRAN.R-project.org/package=vegan) for the R computing platform (v3.2.1) (www.r-project.org) [64]. To examine differences in the relative abundance of dominant groups of the bacterial (genus level) or fungal (family level) community among *stages 1–4*, the Kruskal–Wallis test followed by Tukey’s HSD (honest significant difference) post hoc test was used, both implemented in R with the “agricolae” package (https://CRAN.R-project.org/package=agricolae).

To gauge the relevance of soil physiochemical properties (i.e., *D*_*1*_, *D*_*2*_, *D*_*3*_, *pH*, *AN*, *TN*, *TK*, *TP*, *WST*, *AK*, *SOM*, and *AP*) and assess their ability to explain variation in the distribution patterns of microbial community members in the different biocrusts samples, distance-based redundancy analysis (db-RDA) and Monte Carlo permutations were used. Mantel tests were implemented to evaluate how bacterial or fungal community composition was related to the measured site-level soil variables. Pearson’s *r* coefficient was used to test for a positive (*R* > 0) or negative (*R* < 0) linear correlation between two variables, carried out in R using its cor () function. The “vegan” package was used to run the db-RDA and Mantel tests in R, and also to build the matrices for the pairwise taxonomic distances for bacterial or fungal communities (Bray–Curtis dissimilarity) vis-à-vis the environmental variables (Euclidean distance).

## Results and Discussion

### Response of Soil Physicochemical Properties to Biocrust Succession

With the succession of biocrusts, significant differences in soil properties emerged in moss crust vis-à-vis the other two crust types and the bare sand (i.e., the control) (Table 1). Regarding the three soil particle diameter (*D*) properties—0.002 < *D*_*1*_ ≤ 0.02 mm; 0.02 < *D*_*2*_ ≤ 2 mm; *D*_*3*_ < 0.002 mm—in comparison to bare sand (*stage 1*), there was a lower proportion of *D*_*1*_ in all three biocrust types, which declined through their succession trajectory (*stage 2* > *stage 3* > *stage 4*. Conversely, the *D*_*3*_ proportion rose in all three biocrusts, but their *D*_*2*_ proportion remained similar along the successional gradient. Of the nine soil chemical properties, the contents of seven (*AN*, *TN*, *TP*, *WST*, *AK*, *SOM*, and *AP*) tended to increase in the three biocrust types relative to bare sand, being significantly higher in moss crust (*stage 4*) than either cyanobacterial crust (*stage 2*) or lichen crust (*stage 3*). Impressively, in moss crust, these *AN*, *TN*, *TP*, *WST*, *AK*, *SOM*, and *AP* contents were respectively 3.26, 10, 2.71, 3.38, 5.51, 13.31, and 4.33 times greater than in bare sand, respectively (Table 1). Evidently, the development and succession of biocrusts resulted in the enrichment of the shallow soil layer with carbon (C), nitrogen (N), and phosphorus (P), the most common limiting elements in terrestrial ecosystems. In contrast, both soil *pH* and *TK* were negligibly affected by the succession of biocrusts in Hulun Buir Sandy Land.

**Table 1:** Physicochemical properties of soil without and with different biocrusts, these forming a successional gradient, in Hulun Buir Sandy Land, China

	D_1_ (%)	D_2_ (%)	D_3_ (%)	pH	AN (mg/kg)	TN (mg/kg)	TK (mg/kg)	TP (mg/kg)	WST (mg/kg)	AK (mg/kg)	SOM (mg/kg)	AP (mg/kg)
Stage 1	20.22 ± 0.59A	71.78 ± 0.59A	8.00 ± 0.0B	6.75 ± 0.06A	19.60 ± 2.06B	100 ± 10B	24.60 × 10^3^ ± 100A	70 ± 0B	420 ± 20B	34.90 ± 1.20B	1.65 ± 0.06B	2.39 ± 0.05B
Stage 2	18.19 ± 0.00AB	69.75 ± 1.17A	12.06 ± 1.17A	6.68 ± 0.04A	25.50 ± 2.74B	220 ± 20B	24.90 × 10^3^ ± 150A	110 ± 10B	430 ± 20B	53.97 ± 2.79B	4.10 ± 0.14B	3.21 ± 0.18B
Stage 3	17.86 ± 0.68BC	73.47 ± 1.35A	8.68 ± 0.68B	6.61 ± 0.03A	25.90 ± 1.39B	270 ± 60B	24.23 × 10^3^ ± 320A	90 ± 10B	670 ± 90B	57.97 ± 8.14B	5.16 ± 0.56B	2.74 ± 0.17B
Stage 4	15.82 ± 0.34C	72.12 ± 0.34A	12.06 ± 0.0A	6.98 ± 0.14A	63.97 ± 9.69A	900 ± 190A	24.73 × 10^3^ ± 290A	190 ± 30A	1420 ± 270A	192.33 ± 24.69A	21.97 ± 5.01B	10.4 ± 2.39A

Values shown are the mean ± SE; for a given parameter, those with different letters (A, B, and C) differed significantly at *P* < 0.05.* D*, soil particle diameter, 0.002 mm < *D*_*1*_ ≤ 0.02 mm; 0.02 mm < *D*_*2*_ ≤ 2 mm; *D*_*3*_ ≤ 0.002 mm

AN, available nitrogen; TN, total nitrogen; AK, available potassium; TK, total potassium; TP, total phosphorus; WST, total content of water-soluble salt; SOM, soil organic matter; Stage 1, bare sand (*n* = 3 plots); Stage 2, cyanobacterial crust (*n* = 3); Stage 3, lichen crust (*n* = 3); Stage 4, moss crust (*n* = 3)

These results can be explained by the powerful ecological enhancement function of biocrusts, which mediate most of the input, transport, and output of matter and energy at the surface boundaries of desert soils. Soil aggregates produced by biocrusts have been shown stabilize soil particles and soil structure [65, 66], thereby altering the ecohydrological processes of desert ecosystems [3] in addition to capturing and retaining resources (e.g., soil, organic matter, seeds, and nutrient-rich dust) [67–70]. Further, biocrusts can bolster soil fertility by fixing atmospheric C and N [71, 72] and releasing it into the subsoil layer, thus contributing to the global C and N cycling [73]. Moreover, biocrusts were also identified as a key component of biogeochemical phosphorus cycling during the pedogenesis of sandy substrates [74, 75]. Overall, our findings suggest biocrust development markedly improves the soil properties of bare sand, with a well-developed crust (i.e., moss crust, end of succession: *stage 4*) having a stronger ameliorating effect than less-developed crusts (cyanobacterial or lichen crusts). Actually, our findings largely agreed with those recently reported for European dunes [76].

### Structure and Succession of the Biocrust Microbial Community

**Fig. 2:**
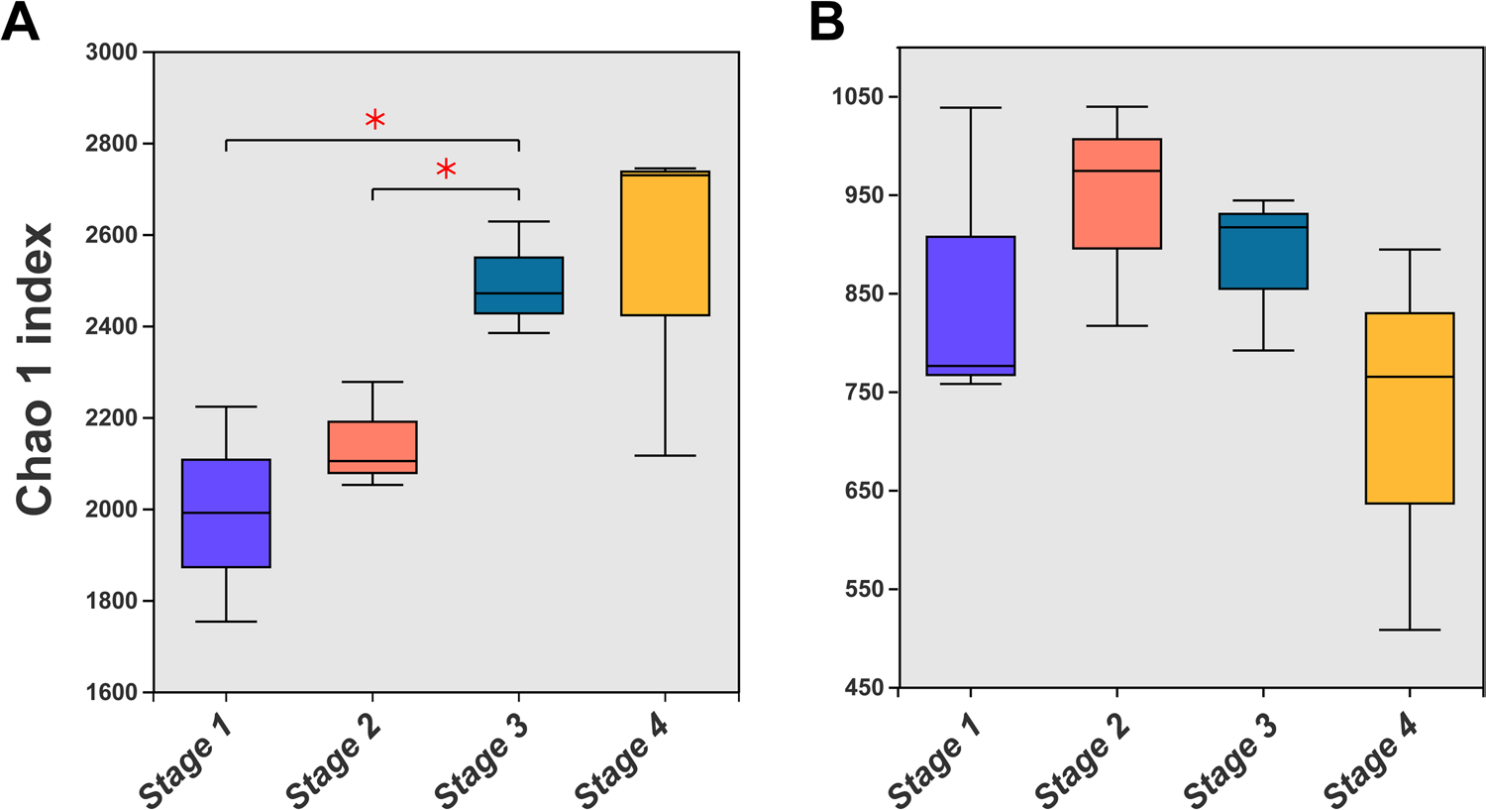
Microbial α-diversity (Chao1 index) of the four biocrust successional types and their comparison. Shown are boxplots for the Chao1 index values of the bacterial (**A**) and fungal (**B**) community in the four stages of biocrust succession. The differences in means between any two were compared statistically by the* t* test. The horizontal line is the median value; an asterisk between two boxplots indicates their significant difference at * *P* < 0.05; *stage 1*, bare sand (*n* = 3); *Stage 2*, cyanobacterial crust (*n* = 3); *stage 3*, lichen crust (*n* = 3); *stage 4*, moss crust (*n* = 3)

Microbial α-diversity was estimated by the Chao1 index (Fig. 2). Whereas its mean value for the bacterial community ranged from 1988.7 ± 235.07 (*stage 1*) to 2529.8 ± 358.53 (*stage 4*) (Fig. 2A), it was much lower for the fungal community, ranging from 722.24 ± 196.56 (*stage 4*) to 943.01 ± 114.2 (*stage 2*) across the successional gradient. In general, while the bacterial community’s α-diversity continually increased, the fungal community’s first increased and then decreased through succession, but these changes were not significant (*P* > 0.05). Other calculated indexes for species richness and diversity of the bacterial and fungal communities are summarized in Table S1.

That bacterial α-diversity increased with biocrust succession—from bare sandy soil to lichen crusts or moss crusts—is consistent with successional theory of low-to-high level shifts in diversity; hence, in this respect, the dynamics of Hulun Buir Sandy Land are much like other desert ecosystems [17, 30, 31, 33, 77–79]. Interestingly, fungal α-diversity was similar across the different stages, conflicting with the view that it continually increases across biocrust successional stages [78, 80]. In general, fungal diversity has been found to vary with both the age and type of biocrusts, being higher in their late than early succession [81]. We suggest this discrepancy is most probably due to habitat specificity effects on a regional scale. Again, our results show that mossy crusts could provide more pivotal resources and protection for soil bacterial communities, mainly because of their higher dust capture, water-holding, and nutrient retention capacities [45].

**Fig. 3:**
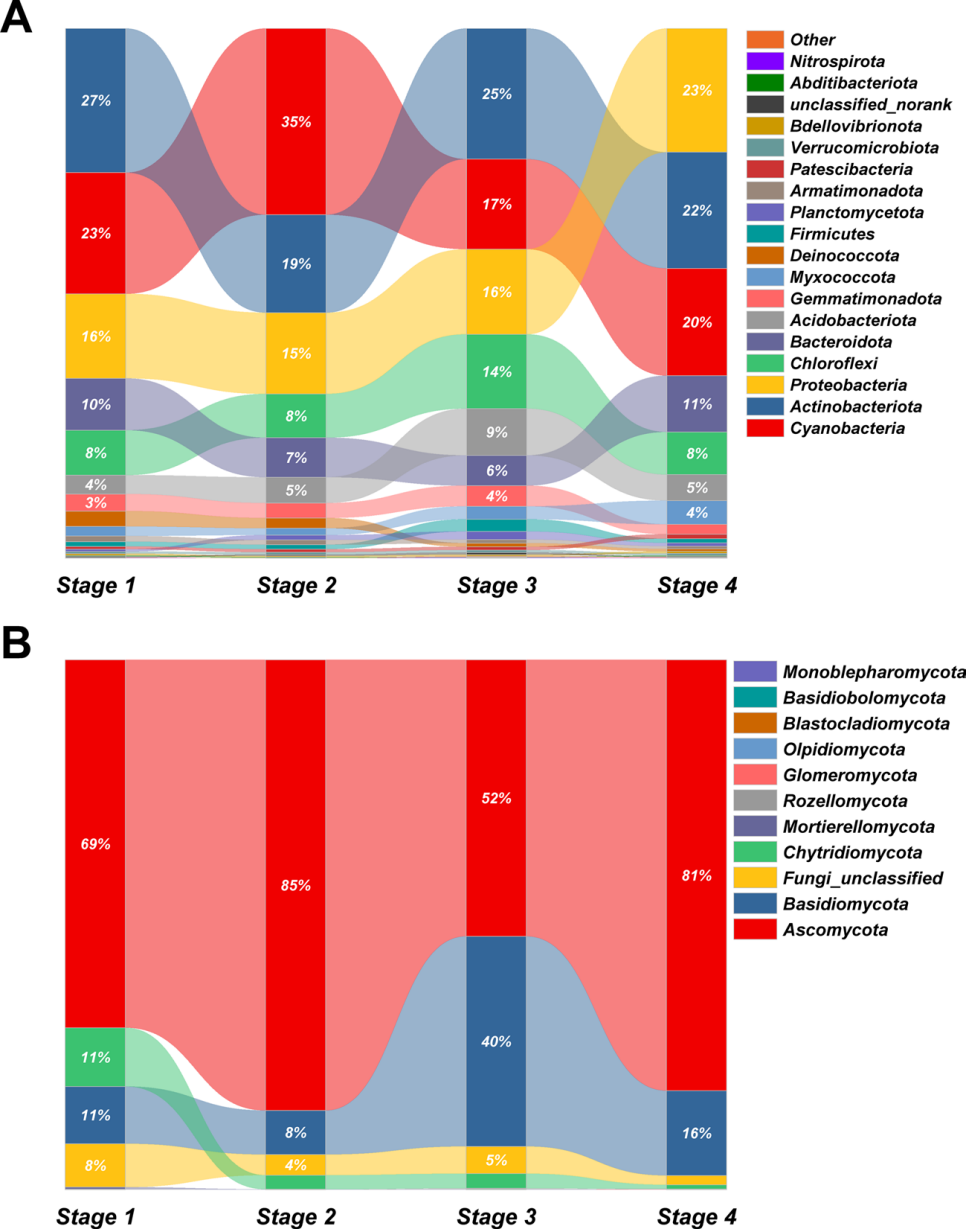
Soil bacterial (**A**) and fungal (**B**) community composition of the four biocrust successional types at the phylum level. *Stage 1*, bare sand (*n* = 3); *stage 2*, cyanobacterial crust (*n* = 3); *stage 3*, lichen crust (*n* = 3); *stage 4*, moss crust (*n* = 3). The percentages are relative abundances

Overall, 32 bacterial and 11 fungal phyla were detected across all 12 plot-level samples based on NGS sequencing. For bacteria, the six dominant phyla (i.e., with a relative abundance > 5%) in all samples of *stages 1–4* were *Cyanobacteria* and *Actinobacteriota*, respectively constituting 23.83% and 23.09% of all sequences, on average; followed by *Proteobacteria* (17.68%), *Chloroflexi* (9.71%), *Bacteroidota* (8.39%), and *Acidobacteriota* (5.53%) (Fig. 3A; Table S2). For fungi, the dominant phylum in all soil samples was *Ascomycota*, on average constituting 72.03% of all sequences, followed far behind by *Basidiomycota* (18.70%), along with Fungi_unclassified (4.73%) and *Chytridiomycota* (4.32%) (Fig. 3B; Table S2). Globally, the bacterial phyla *Actinobacteria*, *Cyanobacteria*, *Proteobacteria*, *Firmicutes*, *Chloroflexi*, *Bacteroidetes*, *Acidobacteria*, *Verrucomicrobia*, *Gemmatimonadetes*, *Planctomycetes*, and *Deinococcus-Thermus* [21, 38, 42, 82] and the fungal phyla *Ascomycota*, *Basidiomycota*, and *Chytridiomycota* [17, 42, 83] have been reported as the most abundant taxa across all biocrusts developmental stages in various desert ecosystems. Hence, our findings strongly agreed with those reported assessments.

**Fig. 4:**
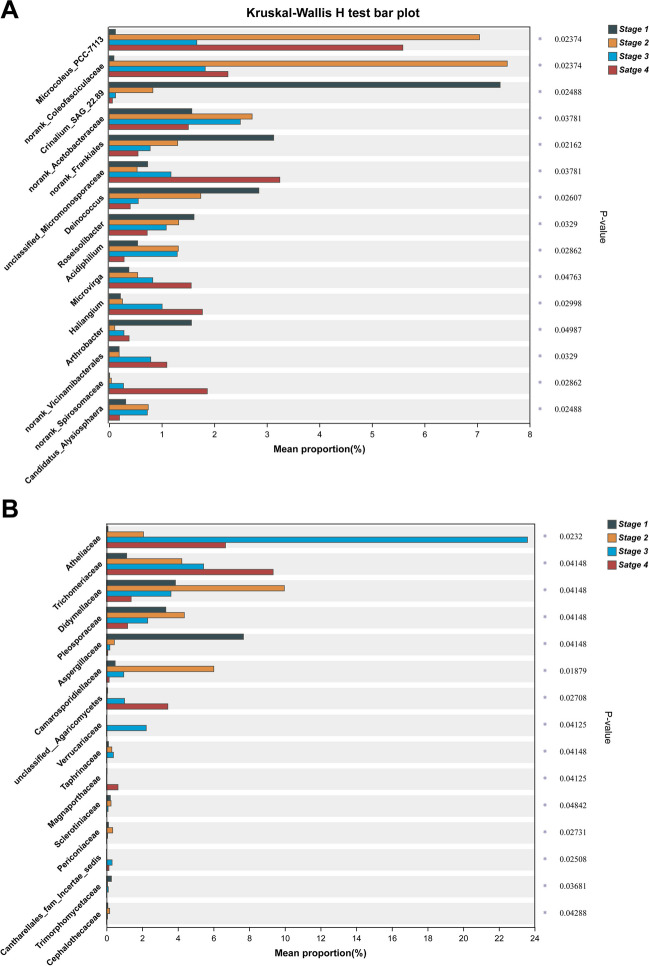
Differences in the relative abundance (%) of the top-15 bacterial genera (**A**) and the top-15 fungal families (**B**) in the microbial communities among the four biocrust successional types. For each taxon, the asterisk on the right indicates a significant difference among stages (*P* < 0.05). *Stage 1*, bare sand (*n* = 3); *stage 2*, cyanobacterial crust (*n* = 3); *stage 3*, lichen crust (*n* = 3); *stage 4*, moss crust (*n* = 3)

Not surprisingly, in our study, the relative abundances of those phyla changed with the successional stage of biocrusts. For bacteria, in shifting from cyanobacterial crust to lichen crust and then to moss crust, the corresponding relative abundance of *Proteobacteria* increased significantly (from 15.33 to 16.08% and then to 23.34%), while that of *Cyanobacteria* decreased significantly (from 35.16 to 17.00% and then 20.23%) (Fig. 3A; Table S2). Meanwhile, a hump-shaped response whereby relative abundance rose then fell was found for *Actinobacteria* (from 18.50 to 24.68% and then 21.98%), *Chloroflexi* (from 8.26 to 14.04% and then 8.06%), and *Acidobacteria* (from 4.89 to 8.78% and then 4.92%). Of them, *Proteobacteria* are dominant in a wide range of harsh conditions, especially oligotrophic habitats, with *Actinobacteria* also described as a dominant group in desert soils given their ability for filamentous growth, which may effectively mitigate damage from drought, high temperatures, and UV radiation [84–86]. Moreover, as the oldest known photosynthetic autotrophic component of biocrusts, *Cyanobacteria* can survive and rapidly grow in water and nutrient-poor desert soils; the fossilized soil structure of a 2.6-billion-year-old biocrust indicates that it was most likely composed of *Cyanobacteria* members [4, 6]. We found that *Firmicutes* usually attained their highest relative abundance in desert topsoil, but then gradually declined in the course of biocrust succession. Similarly, many other studies have shown that, during the succession of biocrusts, the *Cyanobacteria* initially dominant in the cyanobacterial crust undergo a predictable reduction in abundance as *Actinobacteria*, *Proteobacteria*, *Chloroflexi*, *Acidobacteria*, *Gemmatimonadetes*, *Bacteroidetes*, *Planctomycetes*, *Verrucomicrobia*, and *Deinococcus-Thermus* become more common [12–17, 19, 21, 38, 42]. Furthermore, a total of 154 bacterial genera displayed significant differences in their relative abundance across the successional gradient (i.e., *stages 1–4*) (Fig. 4; Table S3). Among those, the 15 most abundant (in descending rank) were *Microcoleus_*PCC-7113, norank_*Coleofasciculaceae*, *Crinalium_SAG*_22.89, norank_*Acetobacteraceae*, norank_*Frankiales* unclassified_*Micromonosporaceae*, *Deinococcus*, *Roseisolibacter*, *Acidiphilium*, *Microvirga*, *Haliangium*, *Arthrobacter*, norank_*Vicinamibacterales*, norank_*Spirosomaceae*, and *Candidatus_Alysiosphaera* (Fig. 4A; Table S3). Both *Microcoleus*_PCC-7113 and norank_*Coleofasciculaceae*, which were always affiliated with *Cyanobacteria*, each reached a significantly higher relative abundance in cyanobacterial crust than in either lichen crust or moss crust and especially vis-à-vis bare sand (*stage 1*) in the Hulun Buir Sandy Land. Although more than 320 cyanobacterial species from 70 genera have been identified in biocrusts so far, few actually participate in biocrust formation [3]. Among these, *Microcoleus* is the most dominant cyanobacterial genus in biocrusts found in most arid and semi-arid regions, such as the Colorado Plateau in the USA [34], the Negev Desert in Israel [46, 47], and both the Gurbantunggut Desert and Tengger Desert in China, as well as the Kyzyl-Kum desert in Uzbekistan [41, 87]; its species are typical filamentous nonheterocystous cyanobacteria. Notably, *M. vaginatus* and *M. steenstrupii* are often affiliated with *Microcoleus*, albeit harboring different adaptations to temperature, and both species appear dominant in cyanobacterial biocrust communities worldwide; the former being more abundant in cooler environments, while the latter dominates warmer ones [3, 41, 88]. Also belonging to the *Cyanobacteria* is the *Coleofasciculaceae* family, whose members reach substantially higher relative abundances in cyanobacterial crust than other biocrust types, being widely found in the Tengger Desert and Kyzyl-Kum Desert, as well as the Tabernas Desert in Spain [39, 41].

Unlike bacteria, for fungi its community composition has been reported to change negligibly during biocrust succession, with *Chytridiomycota* found at lower relative abundance in bare sandy soils [42] whereas *Ascomycota* reached maximal abundances (over 60%) in different successional stages [17]. In our study, however, the relative abundance of *Ascomycota* in the lichen crust (*stage 2*) was below 60% (ca. 52%), while that of *Basidiomycota* reached as high as 40% (Fig. 3B). Conversely, *Chytridiomycota* was greatly reduced in abundance, from 11.08% (bare sand) to 0.79% (moss crust), across the biocrust successional gradient of Hulun Buir Sandy Land. In fact, *Chytridiomycota* has only been detected at very tiny abundances in the Oman and Chihuahuan deserts up to date worldwide [89, 90]. These results are consistent with those of previous studies, which together suggest that *Chytridiomycota* dominate the early stage of biocrust development, hinting at their tolerance of stressful environments [42]. Collectively, these phyla showed no site-specificity and were ubiquitous in previous research in various desert soil and biocrusts. We found a total of 25 fungal families whose relative abundance differed significantly among *stages 1–4* that formed the successional gradient (Table S3). Of those, the 15 most abundant (in descending order) were *Atheliaceae*, *Trichomeriaceae*, *Didymellaceae*, *Pleosporaceae*, *Aspergillaceae*, *Camarosporidiellaceae*, unclassified_*Agaricomycetes*, *Verrucariaceae*, *Taphrinaceae*, *Magnaporthaceae*, *Sclerotiniaceae*, *Periconiacea*e, *Cantharellales_fam_Incertae_sedis*, *Trimorphomycetaceae*, and *Cephalothecaceae* (Fig. 4B; Table S3). This provides compelling evidence that fungal community composition varies more considerably at the family than phylum level through the succession of biocrusts.

### Environmental Factors Influencing the Community Composition of Biocrust Types

**Fig. 5:**
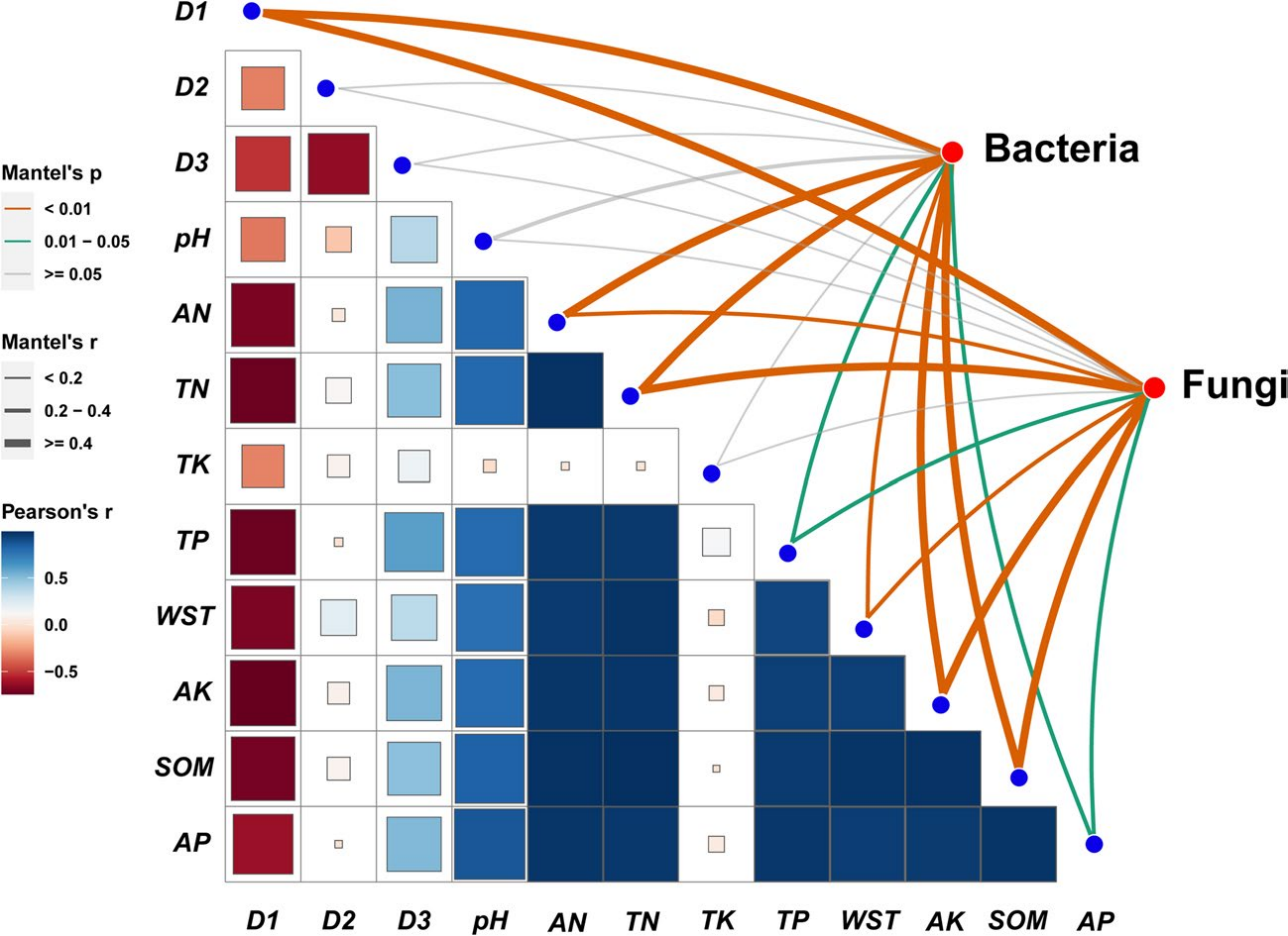
Pairwise comparisons of environmental factors with soil bacterial or fungal community composition of the four biocrusts (pooled, *n* = 12). In the heat map, the magnitude of positive and negative correlations is respectively indicated by the depth of blue and red coloring; Pearson’s *r* > 0 is a positive correlation and *r* < 0 is a negative correlation. The correlation strength (Mantel’s *r*) between bacterial or fungal community composition and a particular environmental factor is indicated by the thickness of their connecting curved line; its color denotes the degree of statistical significance (Mantel’s *p*). The size of each inner box is proportional to Mantel’s *r* statistic for the corresponding distance correlations, whose color denotes the degree of statistical significance (based on 9999 permutations). *D*, soil particle diameter: 0.002 mm < *D*_*1*_ ≤ 0.02 mm; 0.02 mm < *D*_*2*_ ≤ 2 mm; *D*_*3*_ ≤ 0.002 mm; AN, available nitrogen; TN, total nitrogen; AK, available potassium; TK, total potassium; TP, total phosphorus; WST, total content of water-soluble salt; SOM, soil organic matter

The Mantel test results revealed that variation in bacterial and fungal community composition (weighted UniFrac distance matrix-based) responded to the 12 soil parameters examined (Fig. 5; Table S4). Notably, bacterial community composition was positively and strongly correlated with both *D*_*1*_ (*r* = 0.657, *p* = 0.001) and *AK* (*r* = 0.534, *p* = 0.004), moderately so with *SOM* (*r* = 0.454, *p* = 0.003), *TN* (*r* = 0.439, *p* = 0.007), *AN* (*r* = 0.437, *p* = 0.007), and *WST* (*r* = 0.399, *p* = 0.01), and likewise, but to a lesser degree, with *TP* (*r* = 0.375, *p* = 0.016) and *AP* (*r* = 0.335, *p* = 0.023) (Fig. 5; Table S4). The fungal community composition also had positive correlations of similar magnitude with *D*_*1*_ (*r* = 0.715, *p* = 0.001), *AK* (*r* = 0.507, *p* = 0.001), *SOM* (*r* = 0.449, *p* = 0.002), *TN* (*r* = 0.430, *p* = 0.001), *AN* (*r* = 0.394, *p* = 0.006), and *WST* (*r* = 0.389, *p* = 0.01), along with *TP* (*r* = 0.390, *p* = 0.012) as well as *AP* (*r* = 0.342, *p* = 0.043) (Fig. 5; Table S4).

**Fig. 6:**
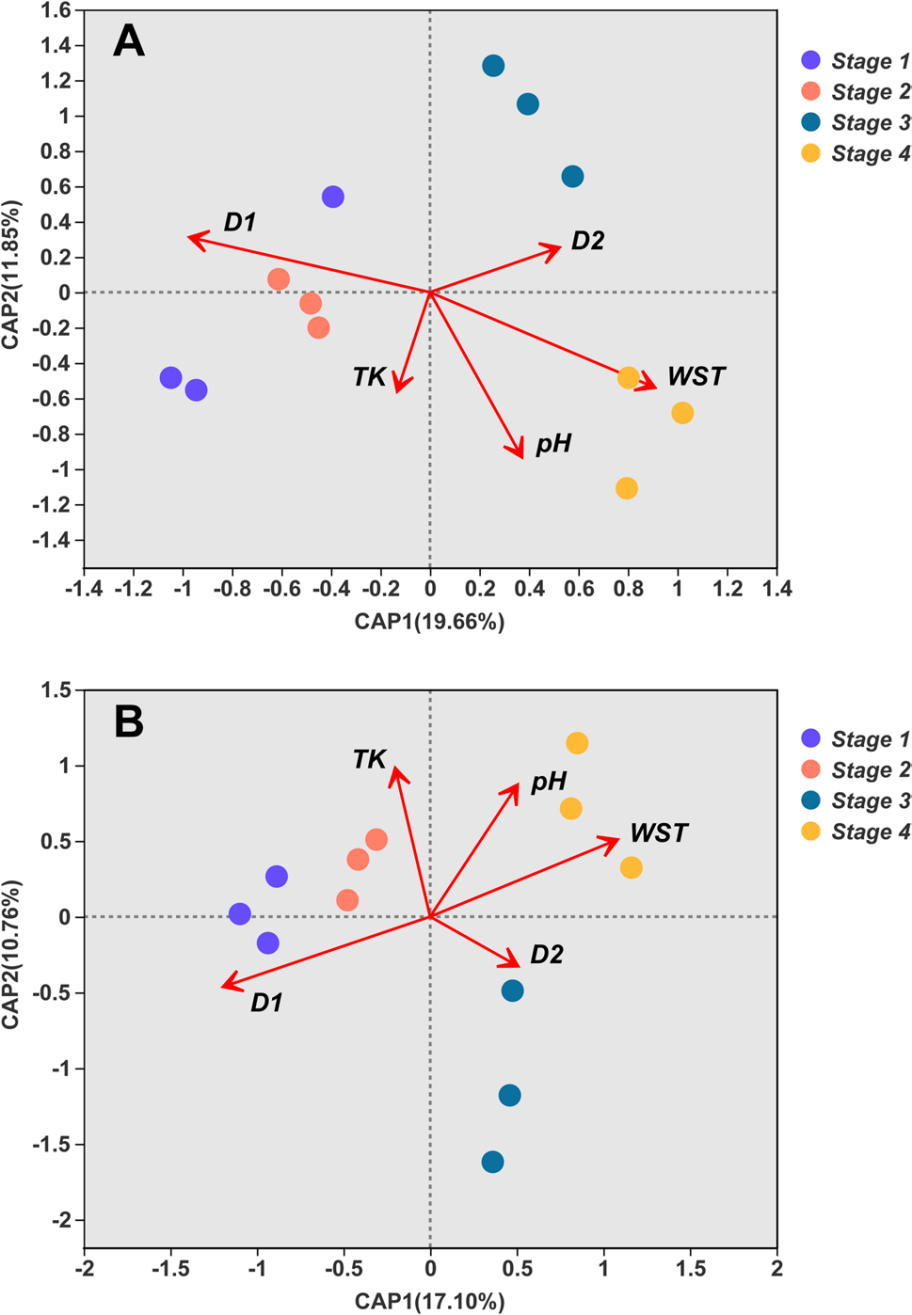
Distance-based redundancy analysis (db-RDA) ordination plots of the relation between five environmental factors and the soil (**A**) bacterial or (**B**) fungal communities of the four successional biocrust types. *D*, soil particle diameter: 0.002 mm < *D*_*1*_ ≤ 0.02 mm; 0.02 mm < *D*_*2*_ ≤ 2 mm; TK, total potassium; WST, total content of water-soluble salt; *stage 1*, bare sand (*n* = 3); *stage 2*, cyanobacterial crust (*n* = 3); *stage 3*, lichen crust (*n* = 3); *stage 4*, moss crust (*n* = 3). Each circular symbol is a plot-level sample

Furthermore, we used db-RDA to evaluate the effects of five VIFs (variance inflation factors) on soil bacterial and fungal community composition (Fig. 6; Table S5). These results showed that about 31.51% of the variance in bacterial community composition could be explained by the selected edaphic properties (Fig. 6A; *CAP1* and *CAP2* explained 19.66% and 11.85% of the variance, respectively). Crucially, three variables alone were mainly responsible for successional shifts in the bacterial community composition of biocrusts: *WST* (*r*^*2*^ = 0.825,* p* = 0.002), *D*_*1*_ (*r*^*2*^ = 0.770,* p* = 0.002), and *pH* (*r*^*2*^ = 0.726, *p* = 0.004) (Fig. 6A; Table S5). Likewise, for fungal community composition, edaphic properties accounted for about 27.86% of its variance (Fig. 6B; *CAP1* and *CAP2* explained 17.10% and 10.76% of the variance, respectively). In this respect, the observed shifts in fungal community composition were driven by four variables: *D*_*1*_ (*r*^*2*^ = 0.868, *p* = 0.001), *WST* (*r*^*2*^ = 0.751, *p* = 0.003), *pH* (*r*^*2*^ = 0.521, *p* = 0.028), and *TK* (*r*^*2*^ = 0.525, *p* = 0.037) (Fig. 6B; Table S5). Nonnegligible, only a small proportion of their community-level variation could be explained by all variables we examined, especially for fungal taxa, for which a high proportion of variation was unexplained. It is largely ascribed to the unmeasured environmental variables. So much unexplained variation in the communities of bacteria and fungi belowground suggested potential effects of neutral or stochastic processes upon community assembly during the succession of biological soil crusts, especially for the fungi [91, 92]. Therefore, more environmental variables, especially availability of soil nutrients (i.e., Ca^2+^, Mg^2+^, and Al^−^), should be incorporated into coupling analysis in the future.

Previous studies have demonstrated that certain soil properties, namely pH, soil organic carbon, and salinity, can variously play an instrumental role in shaping soil microbial diversity and community composition. Thus, as our results suggest, biocrusts may indirectly affect the microbial community in their underlying soil via their modulation of chemical soil properties. Importantly, the impact of environmental factors on soil bacterial and fungal communities depends on the spatial scale considered. Globally, soil pH is deemed the paramount determinant of bacterial community composition [93, 94]. Regionally, however, the soil type, texture, nutrient content, salinity, and moisture are all critical factors governing the bacterial structure and composition of biocrusts [24, 29, 76]. Despite this new knowledge of changing microbial characteristics through the succession of biocrusts, the responsible mechanisms remain unclear. Therefore, distinguishing the fundamental ecological processes (deterministic versus stochastic) shaping soil microbial community composition in the Hulun Buir Sandy Land is a future research priority of ours. Moreover, we used a “space-for-time substitution” sampling approach to reflect the changes in microbial composition along the cyanobacterial crust–lichen–moss crust successional gradient in Hulun Buir Sandy Land. Admittedly, in our analysis, only three samples from each stage included. Consequently, such small sample size may lead to a bias in the analysis of the results. Thus, it is necessary to collect further samples for a complementary analysis in the future.

Over all, our findings thus lend support to this emerging view, and point to complex, possibly divergent mechanisms at work in shaping the successional microbial dynamics of biocrusts in cold desert ecosystems.

## Conclusion

This study employed a “space-for-time substitution” to infer changes in soil properties and microbial dynamics during the succession of biocrusts in the Hulun Buir Sandy Land of Northeast China. Our results revealed significant improvement in the aggregated structure and nutrient status of the shallow soil layer in the course of biocrust succession (i.e., going from bare sandy surface to cyanobacterial crust, then to lichen crust, and eventually to moss crust). Meanwhile, soil bacteria and fungi exhibited contrasting trends during succession, with the former increasing but the latter decreasing. As biocrust succession progressed, soil bacterial and fungal communities at various taxonomic levels (phylum and genus) underwent predictable shifts, albeit to varying degrees, this largely driven by altered soil properties. Although more in-depth studies are needed for the present study, these results still provide guidance for analyzing the ecological functions of biocrusts and related applications.

### Supplementary Information

Below is the link to the electronic supplementary material.

Supplementary file1 (XLSX 40 KB)

## Data Availability

The dataset generated during and/or analyzed during the current study is available at NCBI Sequence Read Archive (SRA) (accession number: PRJNA1026485).

## Abbreviations

*D*: Soil particle diameter, 0.002 mm < *D*_*1*_ ≤ 0.02 mm, 0.02 mm < *D*_*2*_ ≤ 2 mm; *D*_*3*_ < 0.002 mm
AN: Available nitrogen
TN: Total nitrogen
AK: Available potassium
TK: Total potassium
TP: Total phosphorus
WST: Total content of water-soluble salt
SOM: Soil organic matter
NGS: Next-generation sequencing
